# Bacterial Pyomyositis in the Neck Due to Bacteroides Fragilis

**DOI:** 10.7759/cureus.18232

**Published:** 2021-09-23

**Authors:** Kajal Dalal, Christopher M Hernandez, Evan Sanford, Bundhit Tantiwongkosi, Philip G Chen

**Affiliations:** 1 Otolaryngology, University of Texas Health Science Center, San Antonio, USA; 2 Internal Medicine, University of Texas Health Science Center, San Antonio, USA; 3 Otolaryngology, Cookeville Regional Medical Center, Cookeville, USA; 4 Radiology, University of Texas Health Science Center, San Antonio, USA

**Keywords:** bacterial pyomyositis, bacteroides fragilis, intestinal perforation, deep neck space infection, sepsis

## Abstract

Bacterial pyomyositis is characterized by the formation of abscesses in skeletal muscle groups, frequently in the lower extremities. It is most prevalent in tropical climates and associated with *Staphylococcus aureus*. The presentation of pyomyositis in the neck is rare. We present a unique case of pyomyositis caused by a gut bacterium, *Bacteroides fragilis*. Additionally, the case highlights the difficulty in identifying the cause of spread of the bacteria from the gut to the affected musculature.

A 77-year-old diabetic Caucasian male presented with progressive bilateral neck swelling and limited range of motion of the neck. CT imaging confirmed a peripherally enhancing sternocleidomastoid abscess, right pectoralis major muscle abscess, and a hypodense fluid collection found in the anterior mediastinum and retrosternal space. The patient underwent exploration with incision and drainage of the abscess. Blood and tissue culture-confirmed *Bacteroides fragilis*. Subsequent abdominal CT imaging revealed a perforated ascending colon, which, is thought to be responsible for the source of the bacterium.

Bacterial pyomyositis is a rare condition and if not treated early can lead to sepsis and death. We present a rare case of bacteria from a gastrointestinal (GI) source that has not been reported in the literature. This highlights the importance of a thorough evaluation of a source of infection in patients with pyomyositis, especially in the setting of atypical microbes.

## Introduction

Bacterial pyomyositis is characterized by the formation of abscesses in skeletal muscle groups, more frequently reported in the lower extremities and rarely reported in the neck [1]. The causative organism is commonly *Staphylococcus aureus*. Pyomyositis is more prevalent in tropical climates and less frequently found in temperate climates [2]. In temperate areas, pyomyositis is often associated with immunosuppressive conditions, such as HIV, malignancy, and diabetes mellitus. We report a case of an unusual pathogen, *Bacteroides fragilis*, resulting in pyomyositis of the sternocleidomastoid muscle in an immunocompetent male.

## Case presentation

A 77-year-old male presented to the emergency department at a tertiary care medical center with a one-week history of progressive bilateral neck swelling with limited range of motion. Preceding the neck swelling, he reported a week of cough, fever up to 104°F, odynophagia, nausea/vomiting, and dysphagia. At the urgent care clinic, the patient was diagnosed with presumptive flu and was treated with Tamiflu without improvement in symptoms. The patient also complained of shortness of breath and right shoulder/upper chest pain. He denied any other symptoms.

He had a past medical history of well-controlled, insulin-dependent type II diabetes mellitus and coronary artery disease. He was a former 50 pack per year tobacco user who quit 20 years prior.

Upon examination, the patient was well developed, but appeared ill and uncomfortable. The right neck and sternoclavicular areas were erythematous, indurated, fluctuant, and tender. The right pectoral region was erythematous, indurated, and with deep fluctuance to palpation. There were no palpable left neck masses; however, the left neck was notably tender to palpation without overlying skin changes. Trans-nasal flexible fiberoptic laryngoscopy revealed no masses or lesions in the nasopharynx, nasal cavity, oropharynx, larynx, or hypopharynx.

**Figure 1:**
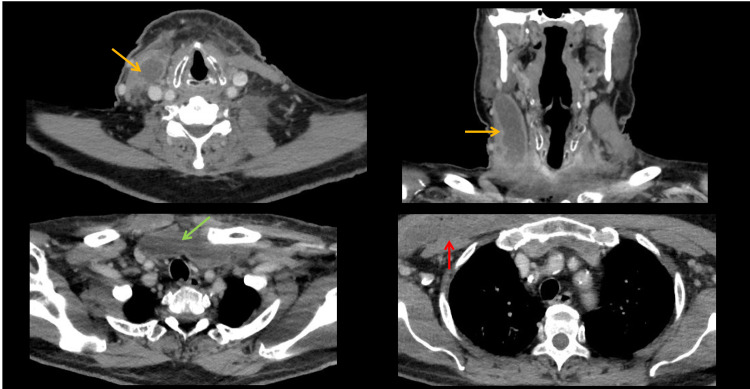
CT neck and chest imaging Orange arrow points to the right sternocleidomastoid abscess. Green arrow points to anterior mediastinal fluid collection. Red arrow points to right pectoralis major abscess.

Initial laboratory workup revealed a leukocyte count of 37,000/mcL (normal value 3400-10,400/mcL) and blood glucose levels ranging between 153 and 199 mg/dL (normal value 60-100 mg/dL). The patient’s most recent hemoglobin A1c (HbA1c) level was 6.8% (normal value <5.7%). Computed tomography (CT) imaging revealed a 7.4 cm peripherally enhancing right sternocleidomastoid (SCM) abscess, a 10.5 cm right pectoralis major muscles abscess, and a 9.2 cm hypodense fluid collection found in the anterior mediastinum and retrosternal space (Figure 1).

A multi-discipline assessment was completed by thoracic surgery, otolaryngology, general surgery, infectious disease, and oral maxillofacial surgery, which concluded that there was no odontogenic source of infection. Subsequently, the patient underwent exploration with incision and drainage (I&D) of the right neck abscess, right anterior neck, and right pectoralis major muscle. Twenty milliliters of thick, purulent fluid were expressed from the right sternocleidomastoid (SCM) abscess. The abscess cavity extended superiorly to the level of the mandible, the midline, and inferiorly to the sternal notch.

To cover deep neck space infection, he was first empirically treated with vancomycin, piperacillin-tazobactam, and clindamycin. Blood and tissue cultures revealed *Bacteroides fragilis*. Antibiotics were adjusted based upon susceptibilities to cefepime and metronidazole.

**Figure 2:**
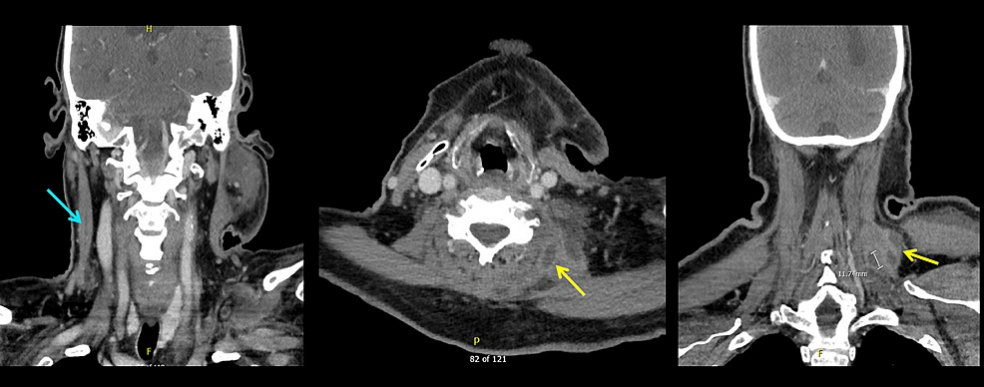
CT neck and chest imaging Blue arrow points to decreased size of right sternocleidomastoid muscle abscess. Yellow arrow points to two new left paraspinal muscle abscesses.

On the second day after surgery for the neck abscess, the patient developed abdominal pain and melena. The patient underwent esophagogastroduodenoscopy revealing a duodenal ulcer with a visible vessel. Abdominal CT imaging revealed pneumoperitoneum, likely arising either from a perforated ascending colon in the hepatic flexure or the perforated duodenal ulcer. On postoperative day three from the neck surgery, the patient underwent an exploratory laparotomy with a right hemicolectomy with a diverting loop ileostomy and a Graham patch of the perforated duodenal ulcer. His abdominal pain was controlled after surgery. However, during this period, repeat neck and chest imaging showed persistent, albeit decreased size of the right sternocleidomastoid muscle abscess, with additional interval development of a left sternocleidomastoid abscess and small abscesses of the left paraspinal muscles (Figure 2).

**Figure 3:**
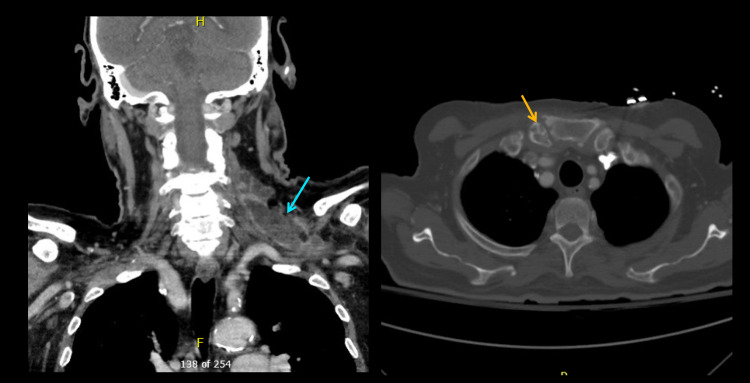
CT neck and chest Blue arrow points to growing left sternocleidomastoid abscess. Orange arrow points to right clavicle osseous erosion.

Following the patient’s hemicolectomy, new non-occlusive venous thromboemboli were discovered in the left and right internal jugular veins, along with a right radial artery dissection and thrombus associated with the patient’s arterial line. He remained inpatient for anticoagulation monitoring and for care following thrombectomy and repair of the right radial artery by vascular surgery. Repeat neck imaging performed one week after initial I&D showed an overall improvement in the abscess involving the sternocleidomastoid/chest. However, two weeks following the initial I&D with ongoing culture-directed antibiotics, CT with contrast revealed an increase in size of the multiloculated abscess involving the left supraclavicular and sternocleidomastoid soft tissues with associated osseous erosion of the medial right clavicle concerning for septic arthritis (Figure 3). On postoperative day 15 from the neck surgery, otolaryngology and cardiothoracic surgery performed an I&D for the new left-sided neck abscess and a washout of the right chest wound. Three drains in total were placed, two were placed deep and posterior to the SCM and one was placed in an abscess pocket posterior to the left clavicular head. Cultures were taken from the new wound and showed no bacterial or fungal growth. The patient also developed new abscesses along the right pectoralis muscle which were irrigated and debrided by general surgery. After several days recovering from surgery, the patient was discharged on postoperative day 25.

Overall, his right sternocleidomastoid abscess and surrounding abscesses significantly improved and the left neck abscess appeared to be healing as well. Interventional radiology was unable to drain any of the remaining fluid collections in his neck.

Two weeks after discharge, the patient returned with concern for a re-perforated duodenal ulcer. CT imaging of the patient’s neck revealed an interval decrease in the size of the multiloculated abscess involving the left sternocleidomastoid muscle and complete resolution of the right pectoral and sternocleidomastoid muscle abscesses. No surgical intervention was indicated. Neck drainage subsided following several days of oral antibiotic treatment. Amoxicillin clavulanate was increased for an additional two weeks to reach a total of eight weeks of antibiotic treatment. The patient was discharged to a rehabilitation facility nine days later. While at the facility, CT imaging demonstrated further decrease in size of the loculated left sternocleidomastoid abscess. Seven months later, after recovering from this hospitalization, the patient returned to the clinic without complaint.

## Discussion

Bacterial pyomyositis is an acute pyogenic infection that results in the formation of single or multiple abscesses in skeletal muscle. It normally affects large muscle group and extremities, such as the quadriceps [1-3]. Presentation of pyomyositis in neck muscles is rare. In the last 20 years, only a few cases have been reported [1,4].

The initiating cause of primary pyomyositis is uncertain; however, it is thought to be a complication of bacterial seeding due to transient bacteremia. Bacteremia alone is normally unable to initiate the formation of intramuscular abscesses [3,5]. Due to the rare nature of pyomyositis, it is assumed that skeletal muscles developed resistance to infection during bacteremic episodes [3,5]. Oftentimes, a preexisting muscle condition, such as trauma or muscle wasting in HIV-infected patients, is thought to facilitate formation of an abscess in the skeletal muscle [1,3,5]. Pyomyositis is more common in immunocompromised patients, such as those with diabetes mellitus, HIV, acquired immunodeficiency syndrome, and autoimmune disease [6]. In this specific case, the patient had a history of type II diabetes mellitus, which could have predisposed him to pyomyositis, yet this patient’s diabetes was well controlled, as evidenced by his HbA1c of 6.8%, and he was otherwise healthy.

*Staphylococcus aureus* is the most commonly cultured organism in cases of pyomyositis. According to a review of reported pyomyositis cases in the last 40 years, *Staphylococcus aureus *was the causative organism of pyomyositis in 70% of cases in HIV-positive patients around the world and 64% of cases in HIV-negative patients in the United States [3,7]. *Staphylococcus aureus* was cultured from pus aspirates of 90% of tropical pyomyositis infections and 75% of non-tropical pyomyositis infections [2,4]. Other common causative pathogens are *Streptococcus​​​​​​​ pyogenes*, other beta-hemolytic Streptococci, *Streptococcus pneumoniae*, *Streptococcus viridans*, *Escherichia coli*, and *Mycobacterium tuberculosis* [2,3]. In the present case, *Bacteroides fragilis* was isolated from the patient's intramuscular abscesses. *Bacteroides fragilis* is an anaerobic, Gram-negative rod found in the colon and is considered the most virulent of its species [8]. Under normal conditions, *Bacteroides fragilis* sustains a favorable relationship with the gut and is mutualistic. When *Bacteroides fragilis* is found outside of the gut, as in cases of GI rupture or GI surgery, it can cause bacteremia and diffuse abscess formation [8]. Some common areas of infection are the abdomen, pelvis, brain, and liver. Since *Bacteroides fragilis* is often found in intestinal mucosal surfaces, damage to mucosal surfaces can allow bacteria to enter the bloodstream [8]. In the present case, the patient had a perforated ascending colon. This damage of GI mucosal barriers may have allowed for* Bacteroides fragilis* to escape and enter the bloodstream and spread hematogenously to the neck, which is rare. As stated above skeletal muscle is resistant to bacterial infection; however, immunosuppression potentially caused by the patient’s type II diabetes mellitus may have increased the patient's susceptibility to intramuscular invasion by* Bacteroides fragilis*.

There are three consecutive stages of pyomyositis. The first stage, also known as the invasive stage, is comprised of an edematous and painful muscle. In this stage, the abscess has not yet formed. There is muscle cramping and progressive pain. The second stage, also known as the suppurative phase, occurs 10 to 21 days later. In this stage, the abscess has formed and there are often high fevers and limited range of motion to affected areas. Edematous muscle fibers and lymphatic infiltration are characteristic during stage two. Most cases of pyomyositis are discovered during this suppurative phase. Lastly, stage three is characterized by systemic symptoms when the patient demonstrates septicemia, widespread abscesses, and multi-organ failure with a high risk of mortality [2,3]. The treatment of pyomyositis is dependent on the disease stage, but generally consists of intravenous antibiotics and abscess drainage [2,3]. Our patient presented with clinical symptoms consistent with that of stage two; he had multiple abscess, a moderately high fever, and limited range of motion of the neck. The patient was initially treated with empiric antibiotics, once the culture results were revealed he was switched to a six-week course of metronidazole.

## Conclusions

In summary, we present a rare pathology of bacterial pyomyositis in a patient with a history of type II diabetes mellitus. This case is unique due to the combination of a rare anatomical location, isolated anaerobic bacterial organism, and the patient’s GI history. This highlights the importance of a thorough evaluation of a source of infection in patients with pyomyositis, especially when atypical microorganisms are identified.
